# Application of signal processing techniques for the spectroscopic analysis of dolutegravir and lamivudine: a comparative assessment and greenness appraisal

**DOI:** 10.1186/s13065-024-01226-y

**Published:** 2024-07-08

**Authors:** Reem M. Alnemari, Ahmed H. Abdelazim, Atiah H. Almalki, Arwa S. Alqahtani, Saleh I. Alaqel, Fahad T. Alsulami, Ahmed Serag

**Affiliations:** 1 Department of Pharmaceutics and Industrial Pharmacy, College of Pharmacy, Taif University, P.O. Box 11099, Taif, 21944 Saudi Arabia; 2 Pharmaceutical Analytical Chemistry Department, Faculty of Pharmacy, Al-Azhar University, Cairo, 11751 Egypt; 3 Department of Pharmaceutical Chemistry, College of Pharmacy, Taif University, P.O. Box 11099, Taif, 21944 Saudi Arabia; 4 Addiction and Neuroscience Research Unit, Health Science Campus, Taif University, P.O. Box 11099, Taif, 21944 Saudi Arabia; 5 Department of Chemistry, College of Science, Imam Mohammad Ibn Saud Islamic University (IMSIU), P.O. Box, 90950, Riyadh, 11623 Saudi Arabia; 6 Department of Pharmaceutical Chemistry, Faculty of Pharmacy, Northern Border University, Rafha, 91911 Saudi Arabia; 7 Department of Clinical Pharmacy, College of Pharmacy, Taif University, P.O. Box 11099, Taif, 21944 Saudi Arabia

**Keywords:** HIV, Dolutegravir, Lamivudine, Spectroscopy, Greenness evaluation

## Abstract

HIV treatment has greatly improved over the years, with the introduction of antiretroviral drugs that target the virus and suppress its replication. Dolutegravir and lamivudine are two such antiretroviral drugs that are commonly used in HIV treatment regimens. Herein, three spectrophotometric methods manipulating ratio spectra were developed for the simultaneous analysis of dolutegravir and lamivudine in their binary mixtures. These methods include mathematical processing stages like ratio difference method or signal processing approaches such as the first derivative of the ratio spectra, and continuous wavelet transform. The developed spectrophotometric methods exploit the characteristic spectral differences between dolutegravir and lamivudine in order to quantify them simultaneously. These methods have shown promising results in terms of sensitivity and selectivity as validated per the ICH guidelines. Moreover, these methods offer a straightforward and economical alternative to more intricate analytical methodologies like high-performance liquid chromatography. By incorporating the analytical eco-scale and AGREE for greenness evaluation of the proposed methods, we can further ensure that these techniques are effective and environmentally friendly, aligning with the principles of green chemistry. This evaluation will provide a comprehensive understanding of the environmental friendliness of these spectrophotometric methods in pharmaceutical analysis.

## Introduction

The use of combination antiretroviral medication has dramatically impacted treatment techniques for those infected with the human immunodeficiency virus (HIV) [1]. There are now around twenty-five antiretroviral medicines classified into six broad groups: protease inhibitors, integrase strand transfer inhibitors, CCR5 antagonists, fusion inhibitors, nucleotide/nucleoside reverse transcriptase inhibitors (NTRI), and non-nucleoside reverse transcriptase inhibitors. Combining nucleotide/nucleoside reverse transcriptase inhibitors with additional drugs is a popular method for HIV therapy [2]. Lamivudine (LMV), an example of an NTRI, has demonstrated success in treating HIV infections and is frequently selected as the initial or subsequent medication choice due to its tolerance and long-term safety record [3]. LMV has recently been utilized to treat HIV patients in conjunction with the integrase inhibitor dolutegravir (DTG) [4]. Clinical investigations have shown that this combination successfully reduces viral loads in those who are infected at high levels. Furthermore, virologic suppression has been achieved and sustained in diverse HIV populations, including those moving from a three-drug regimen, using a two-drug regimen strategy consisting of DTG+LMV [5].

Limited research has been conducted on the analytical methodologies for determining the combination of DTG and LMV in their combined dosage forms. Previous studies have primarily utilized chromatographic separation techniques, including high-performance liquid chromatography (HPLC) and liquid chromatography-tandem mass spectrometry (LC–MS/MS), to analyze these drugs [6–8]. However, it is important to note that these chromatographic methods do possess limitations such as environmentally unfriendly practices due to the significant use of organic solvents and time-consuming optimization processes. UV spectroscopy, on the other hand, offers various advantages, including simplicity, fast analysis durations, and the demand for minimal solvent quantities, making it a more environmentally friendly alternative to chromatographic procedures [9–11]. Furthermore, by merging mathematical manipulation processing stages with spectroscopic analysis, spectrum overlapping concerns may be addressed. The majority of these procedures rely on mathematical formulae or signal processing of the spectra, with no need for chemical or physicochemical separation [12–15]. However, very little literature has reported the application of such methods in DTG and LMV quantitative analysis [16–18]. One of the main drawbacks of these methods is their greenness evaluation as methanol was employed as the main solvent which is known to be hazardous and toxic. Also, none of these methods evaluated some key validation parameters such as the selectivity and specificity of the developed procedures. Besides, some specialized software and experimental designs must be implemented particularly for the chemometric-assisted methods for DTG and LMV analysis.

As a result, the purpose of the current study is to develop sensitive and selective spectrophotometric methods for the analysis of these medications based on mathematical and signal processing procedures manipulating ratio spectra, such as ratio difference (RD), first derivative of the ratio spectra (^1^DD), and continuous wavelet transform (CWT) posing them as powerful alternatives to the reported methods especially in resources limited settings. The suggested techniques will also be evaluated for their greenness using two metrics systems, the analytical eco-scale [19] and greenness metric (AGREE) [20], to ensure their environmental friendliness.

## Experimental

### Materials and solvents

Dolutegravir and lamivudine were provided by the Egyptian Drug Authority (EDA) with purities of 98.7 ± 0.5 and 99.4 ± 0.5, respectively. The dosage form used for analysis was Dovato^®^ tablets (50 mg DTG and 300 mg LMV), manufactured by ViiV Healthcare LLC in the UK. HPLC-grade ethanol was sourced from Sigma-Aldrich in Darmstadt, Germany.

### Instrumentation and software

A Shimadzu UV–Visible 1800 Spectrophotometer was employed for the measurements, with the samples’ absorption spectra being scanned using 10 mm quartz cuvettes. The spectral scan speed was set to fast with bandwidth of 1 nm, and distance between points i.e. data interval of 0.2 nm subsequently, the scanned spectra were obtained in the range of 200–400 nm and analyzed using Shimadzu UV-Probe software version 2.43. The CWT method was implemented in MATLAB R2015a (8.5.0.197613) employing the wavelet toolbox.

### Standard solutions

In order to prepare standard stock solutions for DTG and LMV, 10 mg of each drug powder was separately dissolved in the minimal quantity of ethanol. The resultant solutions were subsequently diluted to a final volume of 100 mL with distilled water using separate 100-mL volumetric flasks. Consequently, both solutions had concentrations of 100 μg/mL.

### Procedures

#### Linearity and calibration graphs

To determine the concentration of DTG and LMV, precise volumes from their working standard solutions were transferred into separate 10-mL volumetric flasks to prepare a series of standard solutions of each drug in the concentration ranges of 1.5–32 and 1.5–36 μg/mL for DTG and LMV, respectively. Distilled water was added to each flask to reach the desired volume. Subsequently, the absorption spectra of these diluted solutions were measured in the wavelength range of 200–400 nm using distilled water as a reference blank. To obtain the ratio spectra of DTG and LMV, the absorption spectra of each drug were divided by a suitable divisor spectrum from the other drug. For DTG, a spectrum of 12 µg/mL LMV was found to be optimal, while for LMV, a spectrum of 18 µg/mL DTG was used.

*For the ratio difference method (RD):* The differences in amplitudes between specific wavelength pairs (258 & 229 nm for DTG and 281 & 241 nm for LMV) in the ratio spectra of each drug were plotted against their corresponding concentrations to create calibration graphs. Linear regression equations were derived from these plots to estimate the concentrations of DTG and LMV accurately.

*For the first derivative of the ratio spectra method (*^*1*^*DD)*: The obtained ratio spectra of each drug were converted to their first-order derivative using a wavelength difference (∆λ) of 4 and a scaling factor of 100. The amplitudes at 271 nm for DTG and at 289 nm for LMV were recorded from the derived derivative spectra. These recorded values were plotted against different concentrations of each drug to construct calibration graphs, from which regression equations were obtained.

*For the continuous wavelet transform method (CWT):* The ratio spectra obtained for each drug were converted to the wavelet domain using the bior 2.4 wavelet family and a scaling factor of 20 under Matlab environment. The amplitudes at 259 nm for DTG and at 281 nm for LMV were recorded from the transformed spectra. The obtained values were plotted against various concentrations of each drug to create calibration graphs, which resulted in the derivation of regression equations.

#### Validation of the developed methods

Validation of the developed methods was conducted following ICH Q2 (R1) guidelines for validation of analytical procedures [21]. Linearity was assessed using nine different DTG and LMV concentrations with the procedures mentioned in Sect. "Linearity and calibration graphs". The resulting calibration curve yielded values for correlation coefficient (R2), slope, and y-intercept. Limits of detection and quantification were determined based on standard deviation of the response, calculated from residuals, and slope of the calibration curve using LOD = 3 × (σ/S) and LOQ = 10 × (σ/S), where σ is the standard deviation of the response and S is the slope.

The accuracy and precision were evaluated by comparing measured concentrations with known ones for three replicates at levels of 6, 12, and 18 µg/mL to determine percentage recovery as well as relative standard deviation (% RSD). To assess the specificity of the developed techniques, different concentrations of DTG and LMV from reference solutions were transferred into separate 10-mL volumetric flasks and diluted with water. The samples were then subjected to analysis using the prescribed protocols for linearity and calibration curves for each drug, enabling computation of their individual concentrations.

#### Greenness evaluation of the developed methods

The greenness evaluation of the proposed methods was conducted based on established criteria for environmentally friendly analytical techniques. Analytical Eco-Scale was used to assess the greenness of the proposed methods based on criteria such as waste generation, energy consumption, and the use of hazardous materials [19]. AGREE prep software was also utilized to evaluate the environmental impact of the proposed methods by considering the 12 principles of green chemistry [20].

#### Analysis of pharmaceutical tablets

To determine the concentration of DTG and LMV, five Dovato^®^ tablets weighing 50 mg DTG and 300 mg LMV each were pulverized into a fine powder. A precise quantity equivalent to 5 mg DTG and 30 mg LMV was then transferred to a 100-mL volumetric flask containing 40 mL of ethanol. After vigorous shaking for 20 min, the mixture was filtered. Additional ethanol was used to adjust the final solution volume as per desired concentration, followed by subsequent dilutions with water resulting in the preparation of five samples with varying concentrations. Each sample underwent analysis following specified procedures as described in linearity assessment and calibration curve plotting method protocols, allowing computational determination of drug concentrations present in the samples.

## Results and discussion

### Spectral characteristic

**Fig. 1:**
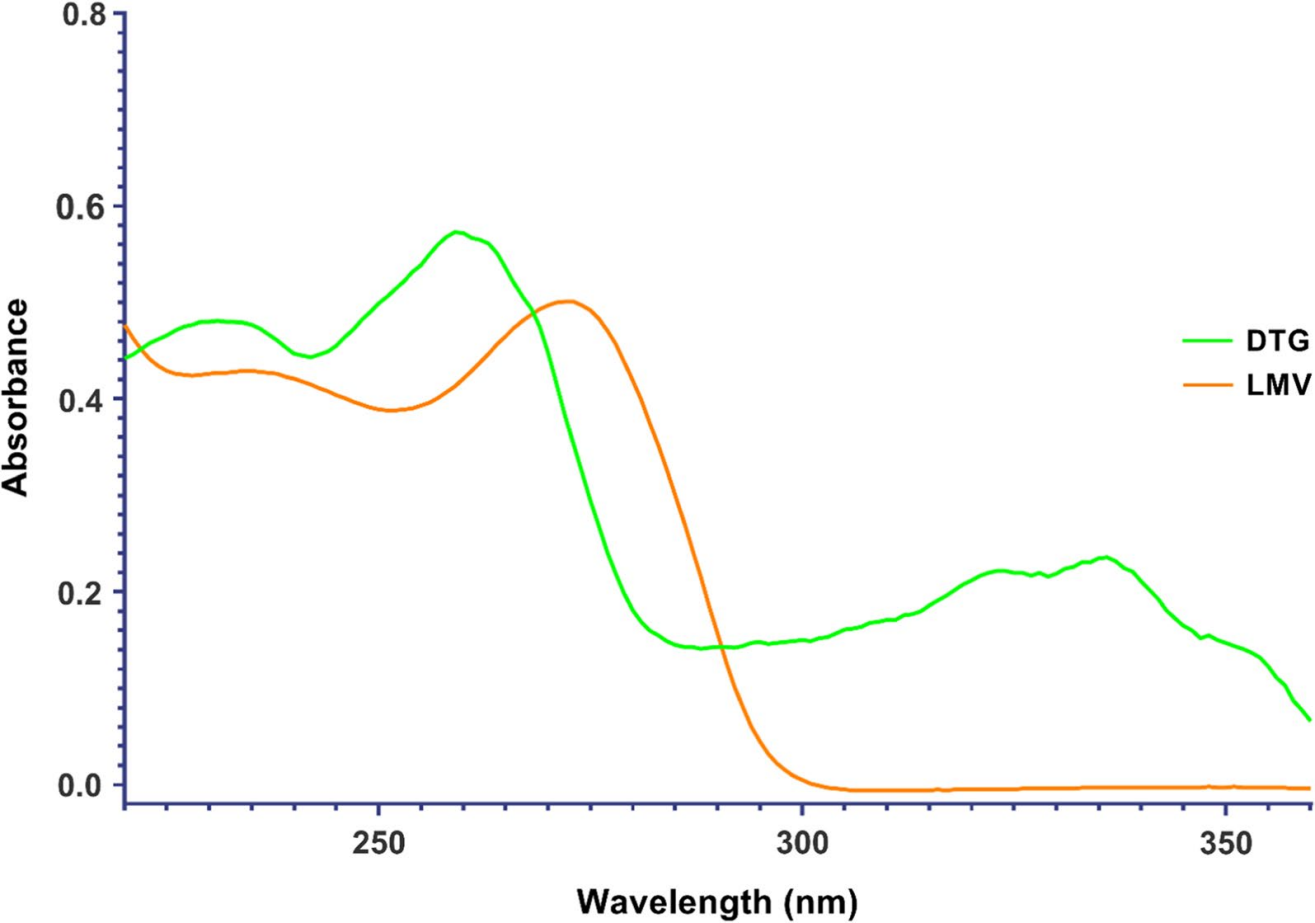
Zero order absorption spectra of 12 μg/mL DTG & 12 μg/mL LMV showing severe overlapping

The UV spectrum of DTG exhibits two significant absorption peaks in the UV region: one at 260 nm and another at 336 nm. These peaks can be linked to the electronic structure of DTG (Fig. 1**)**. Specifically, the peak at 260 nm is likely due to the electronic transitions in the aromatic rings and the other pi-conjugated systems within the compound. Meanwhile, the peak at 340 nm could be attributed to n-pi* transitions, which involve the non-bonding electrons of electronegative atoms of DTG such as oxygen and nitrogen. These functional groups absorb at slightly longer wavelengths than the pi-pi* transitions of aromatic rings. On the Other hand, LMV shows only a single absorption peak in the UV region at around 270 nm (Fig. 1**)**, which can be attributed to the pi-pi* electronic transitions of the cytosine ring—a heterocyclic aromatic ring with both carbon and nitrogen—it’s this part of LMV that is likely responsible for the absorption at 270 nm. As a consequence of these results, DTG and LMV exhibit significant overlap in their absorption spectra at zero-order, making it challenging to accurately determine the concentration of each drug (Fig. 1). In order to overcome this issue, three mathematical spectrophotometric methods have been developed to manipulate their ratio spectra by applying constant removal of the interfering compound *i.e.* the other drug either via mathematical processing techniques like RD method or signal processing approaches such as ^1^DD and CWT. Implementing these preprocessing steps assists in eliminating interfering components without prior separation, thus facilitating the accurate measurement of DTG and LMV in their combined dosage form.

### Methods development and optimization

**Fig. 2:**
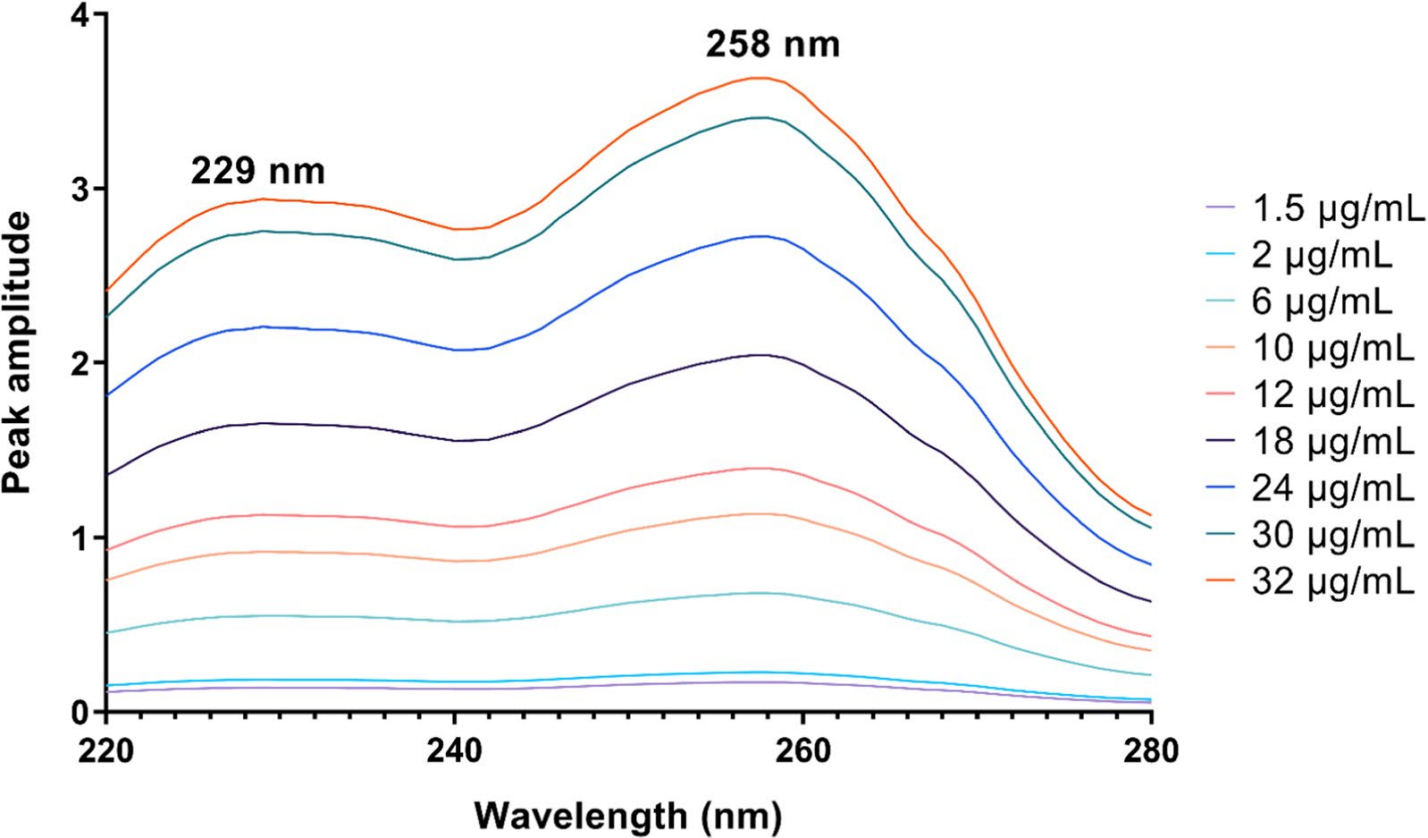
Ratio spectra of DTG in the range of 1.5–32 µg/mL using 12 µg/mL LMV as a divisor

**Fig. 3:**
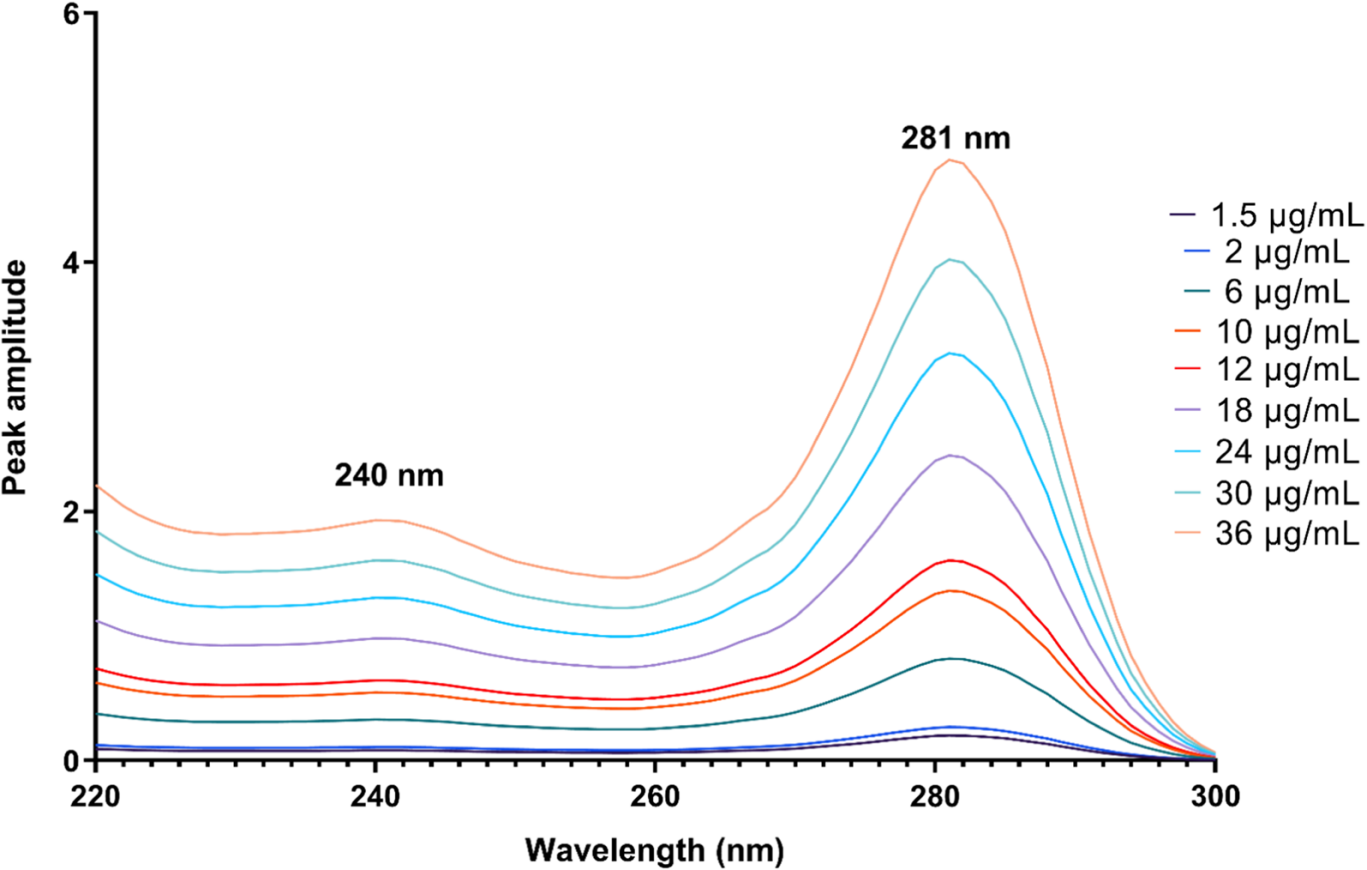
Ratio spectra of LMV in the range of 1.5–36 µg/mL using 18 µg/mL DTG as a divisor

The RD method relies on the use of a mixture containing two drugs, X and Y, with overlapping spectra. To determine the presence of X, the spectrum of the mixture is divided by a known concentration of Y. This division produces a new curve representing X/Y’ and Y/Y’, where Y/Y’ remains constant. By choosing two wavelengths λ1 and λ2 from this ratio spectrum and subtracting their amplitudes, any constant values along with instrumental errors or sample matrix interferences can be eliminated [22]. Hence, this method is influenced by two main factors: the concentration of the divisor and the selection of wavelengths. The concentration of the divisor plays a crucial role in determining the structure of the obtained ratio spectra for DTG and LMV. After careful examination, it was determined that divisors concentrations of 12 μg/mL for LMV and 18 μg/mL for DTG exhibited higher linearity with minimal noise and improved sensitivity. Additionally, choosing appropriate wavelength pairs is vital as it affects important analytical parameters such as intercept, slope, and correlation coefficient in the derived calibration curves. Different wavelength pairs were tested for each drug, revealing that using (258 & 229 nm) for DTG and (281 & 241 nm) for LMV resulted in optimal outcomes, as depicted in Figs. 2 and 3. Then, the regression equations were derived by constructing calibration curves that plotted the difference in amplitude at these specific wavelengths against the corresponding concentrations of DTG and LMV, respectively (Table 1).

**Table 1:** Regression and validation data for quantitative analysis of DTG and LMV by the proposed methods

Parameters	RD		^1^DD		CWT	
	DTG	LMV	DTG	LMV	DTG	LMV
Wavelength (nm)	229 & 258	240 & 281	271	289	259	281
Linearity range (µg/mL)	1.5–32	1.5–36	1.5–32	1.5–36	1.5–32	1.5–36
Slope	0.0216	0.0805	0.5064	1.1566	0.4076	1.418
Intercept	0.0007	0.0063	0.0235	0.0877	0.0142	0.1074
Coefficient of determination (r^2^)	0.9999	0.9999	0.9999	0.9999	0.9999	0.9999
LOD (µg/mL)	0.330	0.477	0.330	0.478	0.358	0.473
LOQ (µg/mL)	1.000	1.445	0.999	1.449	1.085	1.433
Accuracy (%R)^a^	99.61	100.35	98.83	99.99	100.83	99.99
Repeatability precision (%RSD)^b^	0.411	0.347	0.299	0.318	0.693	0.414
Intermediate precision (%RSD)^b^	0.589	0.401	0.351	0.423	0.862	0.736

^a^Average of 9 determinations (3 concentrations repeated 3 times)

^b^RSD of 9 determinations (3 concentrations repeated 3 times)

**Fig. 4:**
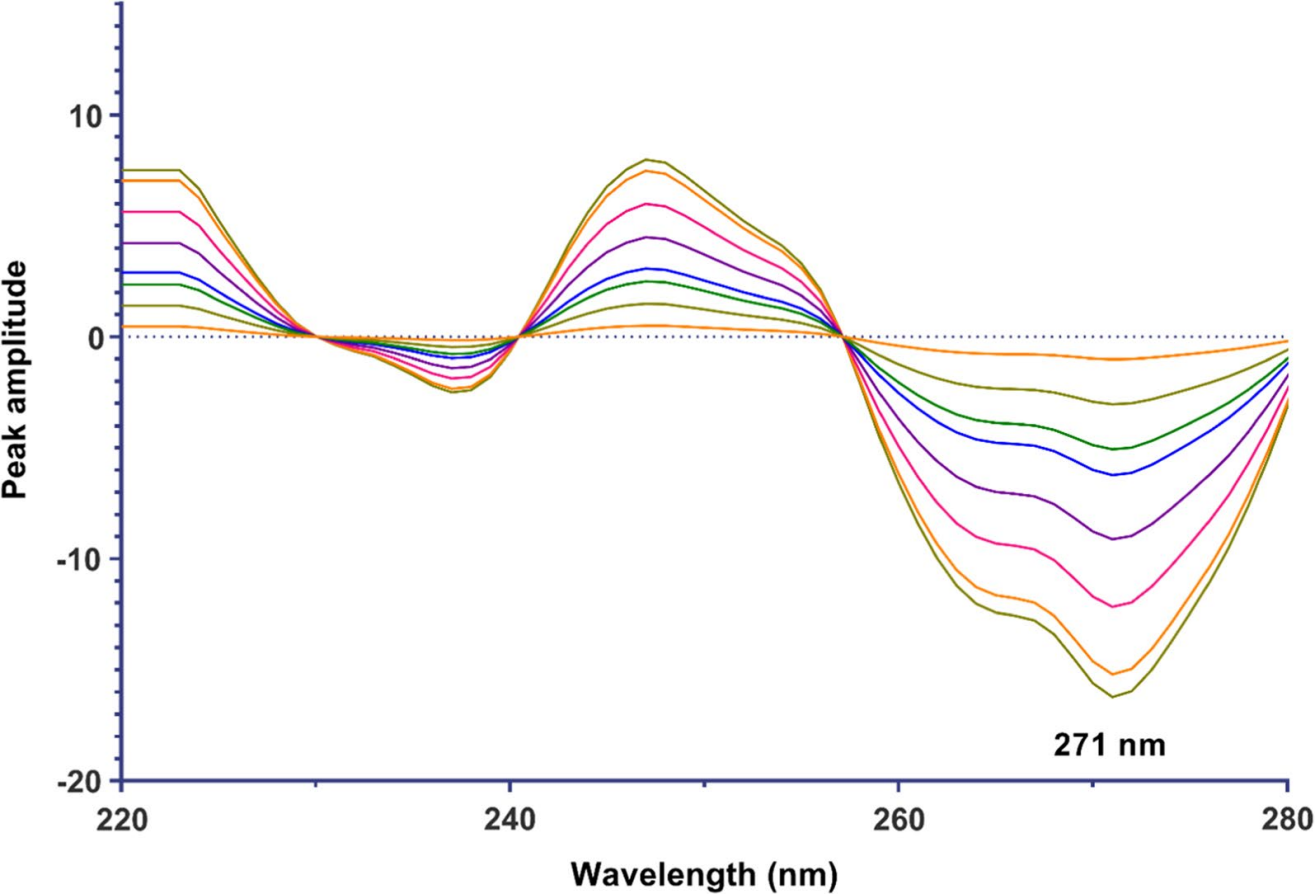
^1^D ratio spectra of DTG in the range of 1.5–32 µg/mL using 12 µg/mL LMV as a divisor

**Fig. 5:**
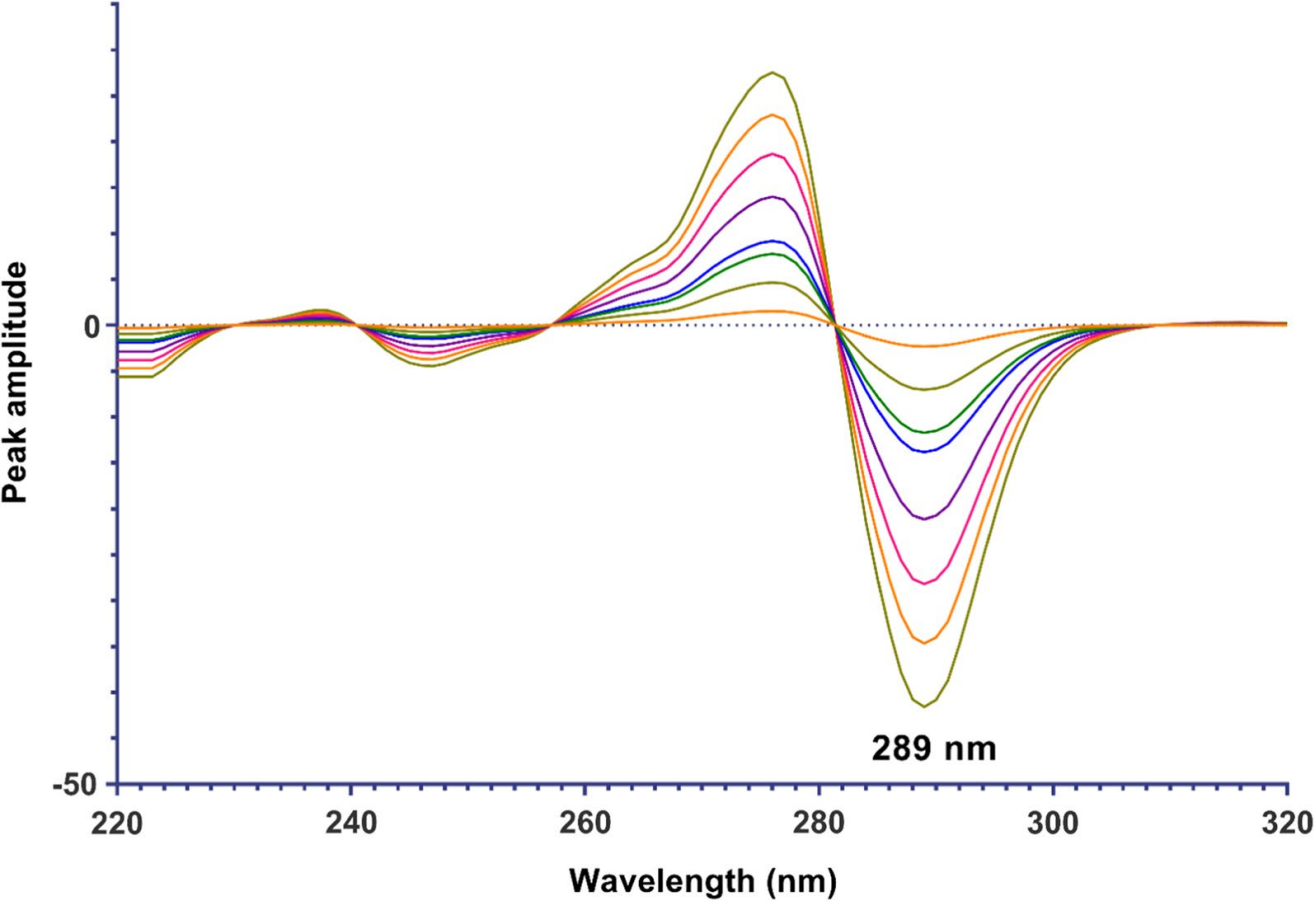
^1^D ratio spectra of LMV in the range of 1.5–36 µg/mL using 18 µg/mL DTG as a divisor

Another method that can be utilized to determine the concentration of DTG and LMV is derivative spectrophotometry, which functions as a signal processing technique. Derivative spectrophotometry is an analytical approach that proves advantageous in resolving spectral overlap by analyzing higher derivatives of absorbance with respect to wavelength. By applying the first derivative to the ratio spectra data of both drugs, it becomes possible to quantify DTG at 271 nm and LMV at 289 nm, as depicted in Figs. 4 and 5. To investigate the influence of instrumental parameters on the formation of derivative spectra, we conducted experiments to explore different smoothing and scaling factors. Our findings revealed that using a Δλ value of 4 nm and a scaling factor of 100 produced optimal results. Subsequently, regression equations were computed based on the first derivative of ratio spectra (^1^DD) for each drug in relation to their final concentrations. These equations are summarized in Table 1.

**Fig. 6:**
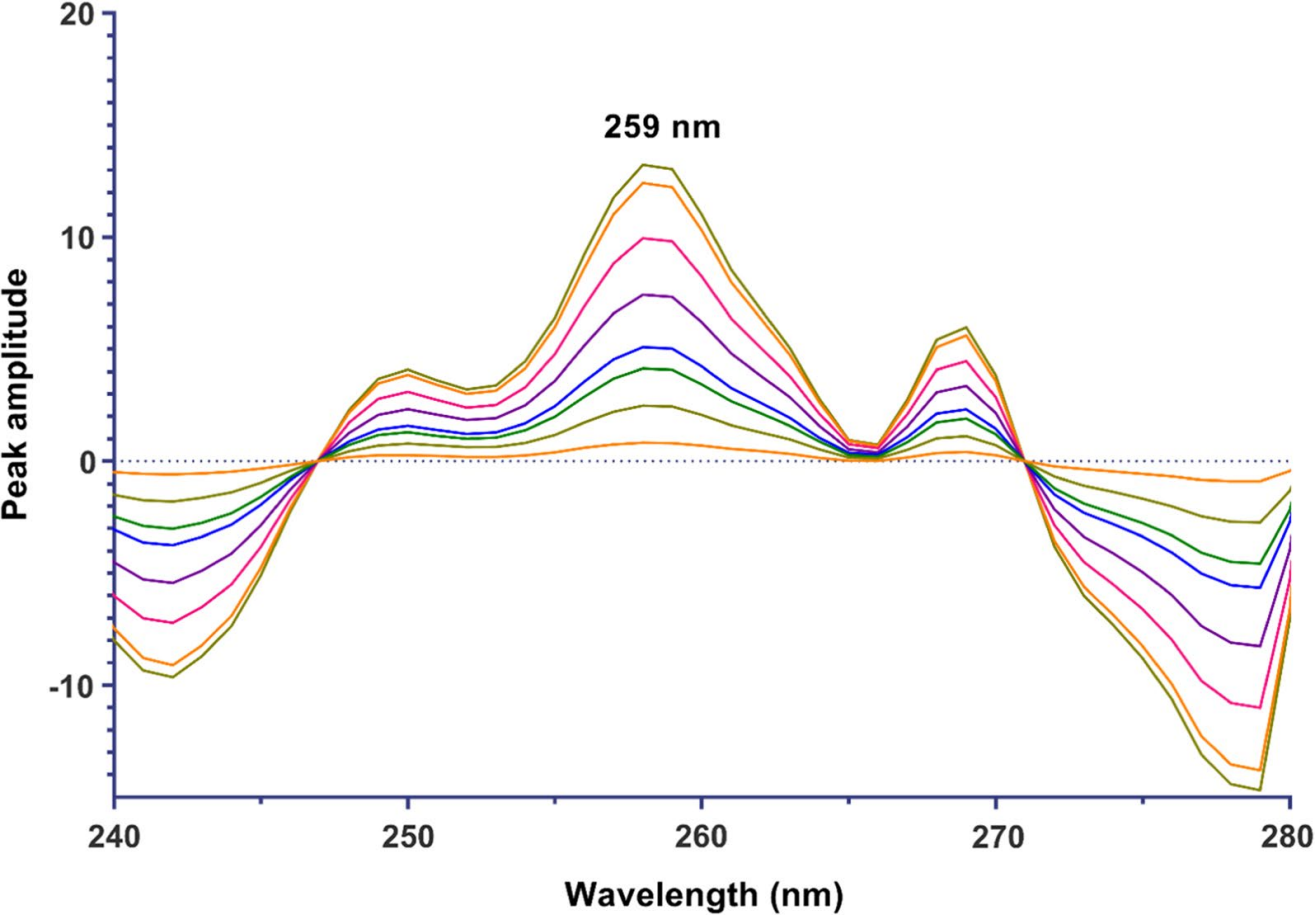
Continuous wavelet transform ratio spectra of DTG in the range of 1.5–32 µg/mL using 12 µg/mL LMV as a divisor

**Fig. 7:**
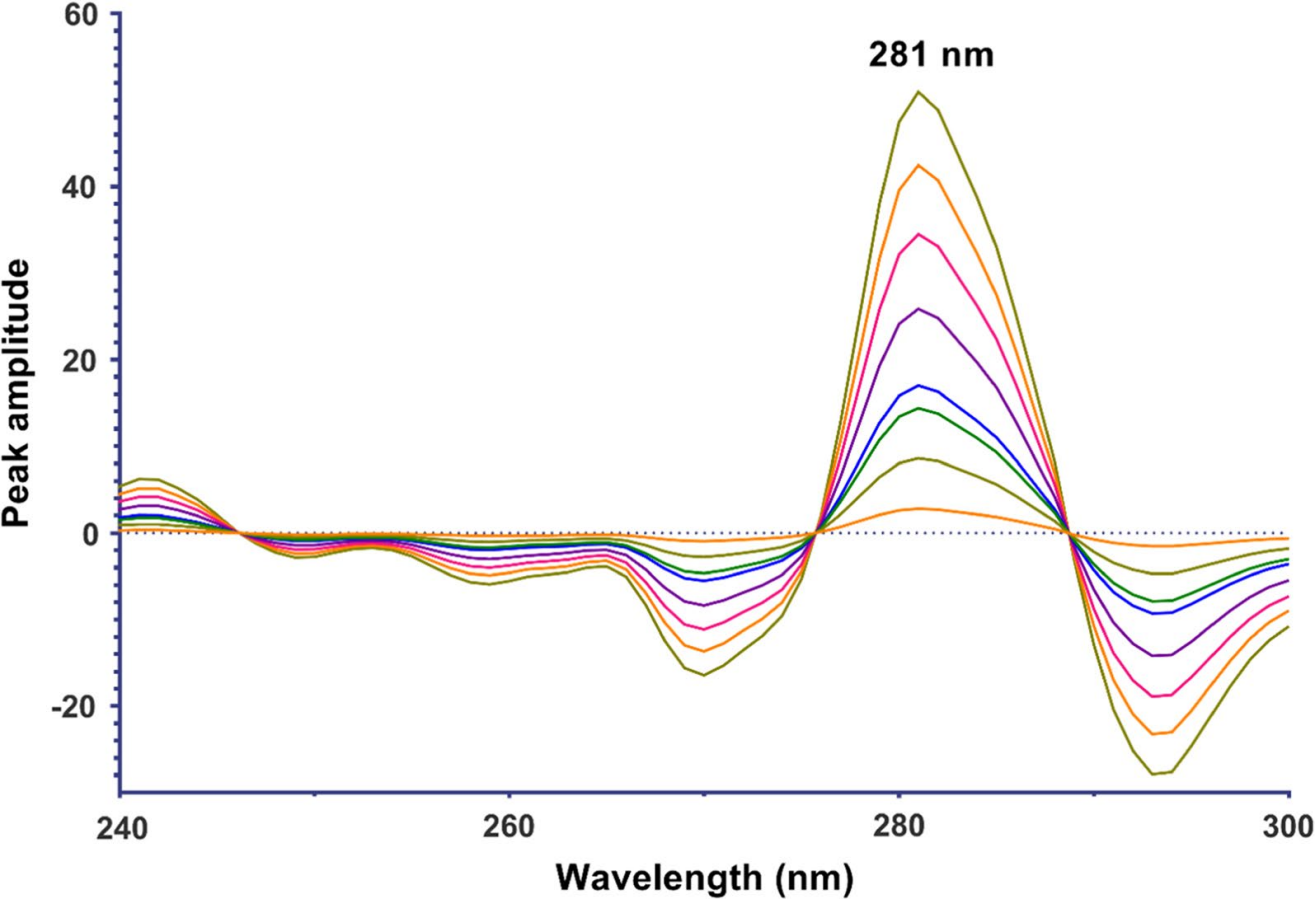
Continuous wavelet transform ratio spectra of LMV in the range of 1.5–36 µg/mL using 18 µg/mL DTG as a divisor

Another signal processing approach is the CWT which was employed for analyzing DTG and LMV in their binary mixture. The wavelet transform represents a powerful mathematical approach used in signal processing and data analysis. Its primary aim is to remove any constant elements or interference from the sample matrix, enabling accurate determination of drug X concentration within a mixture. The fundamental concept involves expressing any arbitrary function as a combination of wavelets, defined as functions Ψa,b (λ) derived from a base function Ψ(λ) through dilation and shift [23]. This base function is often referred to as the “mother” wavelet since it generates a family of wavelets. Various families of wavelet bases exist, such as Daubecies, Symlet, Coiflet, Meyer etc., with the mother wavelet generating functions Ψa,b (λ) by scaling and shifting. The CWT produces multiple coefficients C that are dependent on scale (s) and position (τ). Multiplying each coefficient by appropriately scaled and shifted wavelets yields constituent components from the original signal—a process applicable for decomposing drug mixture spectra to identify specific contributions from each drug. In this context, various wavelet families such as Coiflet (*Coif*), Biorthogonal (*bio*r), Daubechies (*Db*), Gaussian, etc., were tested on the ratio spectra at different orders and with different scaling parameters. The goal was to obtain acceptable calibration curves and ensure accurate determination of DTG and LMV. After thorough testing, it was determined that the *bior* wavelet family of second order (2.2) with a scaling factor (a) of 20 yielded the best results for DTG and LMV analyses. Subsequently, CWT spectra were generated by plotting the obtained CWT-coefficients against wavelengths as shown in Figs. 6 and 7. To construct calibration graphs for DTG and LMV, transformed signals' amplitudes were plotted against their corresponding concentrations at 256 nm and 218 nm, respectively which led to the derivation of the regression equations for both compounds (Table 1).

### Methods validation

Validation of the developed spectrophotometric methods was conducted to assess their accuracy, precision, and selectivity following the ICH guidelines [21]. In this context, the linearity of the calibration curves was evaluated by analyzing a series of standard solutions with known concentrations of DTG and LMV. The results showed that all the developed methods exhibited good linearity over the concentration range 1.5–32 µg/mL and 1.5–36 µg/mL for DTG and LMV, respectively. Additionally, the methods showed high sensitivity exemplified by their low detection limits for DTG ranging from 0.330 to 0.358 µg/mL and for LMV ranging from 0.473 to 0.478 µg/mL, respectively (Table 1**)**. Regarding accuracy, both DTG and LMV showed percent recovery values ranging from 98 to 102%, suggesting that the established methods offer excellent results in accurately determining their concentrations. Moreover, the precision of the developed spectrophotometric methods was evaluated by calculating the percent relative standard deviation (% RSD) of replicate measurements for each drug over several different concentrations. The percentage relative standard deviation (% RSD) values for these methods were found to be within acceptable limits, indicating good precision (Table 1**)**. In addition to linearity, accuracy and precision, the selectivity of the proposed methods was examined by analyzing different laboratory-prepared mixtures with varying ratios of DTG and LMV using the suggested techniques. The findings presented in Table 2 indicate that both drugs can be accurately measured without any interference from each other, thereby confirming satisfactory selectivity.

**Table 2:** Application of the proposed methods for determining DTG and LMV in their laboratory mixed solutions

Added (µg/mL)		%Recovery					
		**RD**		^**1**^**DD**		**CWT**	
DTG	LMV	DTG	LMV	DTG	LMV	DTG	LMV
2	12	100.23	100.07	98.59	99.99	98.71	98.44
3	18	99.23	101.01	98.57	101.00	99.23	100.52
4	24	98.73	100.24	98.02	100.21	98.61	98.41
6	18	99.00	100.32	98.62	100.29	98.85	99.33
8	24	99.71	100.66	99.02	100.61	99.76	100.58
6	6	100.59	99.76	98.82	99.41	99.37	99.27
6	12	101.48	100.89	100.11	99.75	101.15	100.08
8	16	98.24	98.09	101.93	100.98	98.18	100.98
12	6	100.23	100.91	98.93	100.40	98.18	99.63
18	6	101.57	99.22	99.96	99.32	99.18	100.69
24	8	98.9	100.22	101.65	99.69	101.29	100.45
24	4	98.74	100.52	99.71	99.84	100.24	100.68
**Mean ± SD**		99.72 ± 1.102	100.16 ± 0.830	99.49 ± 1.237	100.12 ± 0.558	99.40 ± 1.041	99.92 ± 0.890

### Greenness assessment of the developed methods

The environmental compatibility of the proposed methods in comparison with the reported methods was also assessed using green chemistry principles via implementing two metric approaches, namely analytical eco-scale and AGREE metrics [24]. The analytical eco-scale metric calculates penalty points based on two primary analytical parameters. One parameter is related to the reagents used, which can be estimated by considering their quantities as well as environmental hazards. The other parameter is associated with the instrumentation used, that depends on the energy consumption during operation, and waste generated by the device. After calculating these penalty point scores, the results are subtracted from 100 to determine whether the approach qualifies as outstanding (green), reasonable (semi-green), or inadequate in terms of its environmental sustainability. Using this greenness assessment tool, the developed analytical methods for the quantitative measurement of DTG and LMV in their pharmaceutical preparation were evaluated. The results indicate that the developed methods yielded a score of 3 penalty points in comparison to the reported methods including the LC–MS/MS (22 penalty points), HPLC (19 penalty points), UPLC (18 penalty points) and even the other reported spectrophotometric methods (9 penalty points), suggesting outstanding green analysis of the developed methods (Table 3). Such results could be attributed to the careful consideration of green chemistry principles in the selection of reagents and instrumentation of the developed spectrophotometric methods, leading to reduced environmental impact. On the other hand, most of the reported chromatographic techniques such as LC–MS/MS, HPLC, and UPLC were found to have higher penalty point scores due to excessive use of hazardous reagents such as methanol and acetonitrile in addition to higher energy consumption. The reported spectrophotometric methods also had higher penalty point scores compared to the developed methods, indicating they are less environmentally friendly due to the employment of methanol as a solvent.

**Table 3:** Greenness evaluation and comparison of the proposed method and reported methods using the described metrics

Method	Analytical eco-scale		AGREE tool
	Parameters	Penalty points	
Proposed method	Reagents		
	Water	0	
	Instrument spectrophotometry		
	Energy consumption	0	
	Occupational hazards	0	
	Waste	3	
		**∑**3	
	Total scores	100–3 = 97	
Spectrophotometric methods ^16–18^	Reagents		
	Methanol	6	
	Phosphate buffer pH 6.8	0	
	Water	0	
	Instrument spectrophotometry		
	Energy consumption	0	
	Occupational hazards	0	
	Waste	3	
		**∑**9	
	Total scores	100–9 = 91	
LC–MS/MS ^7^	Reagents		
	Acetonitrile	8	
	Formic acid	6	
	Instrument LC–MS/MS		
	Energy consumption	2	
	Occupational hazards	0	
	Waste	6	
		**∑**22	
	Total scores	100–22 = 78	
UPLC ^8^	Reagents		
	Methanol	12	
	K^+^ dihydrogen orthophosphate	0	
	Instrument UPLC		
	Energy consumption	0	
	Occupational hazards	0	
	Waste	6	
		**∑**18	
	Total scores	100–18 = 82	
HPLC ^6^	Reagents		
	Methanol	12	
	Sodium dihydrogen phosphate	0	
	Instrument HPLC		
	Energy consumption	1	
	Occupational hazards	0	
	Waste	6	
		**∑**19	
	Total scores	100–19 = 81	

The other metric system: AGREE is an easily accessible software tool that adheres to the principles of green analytical chemistry and produces a clock-like graph output based on twelve input criteria, displaying an overall score and color representation, where a perfect analysis would attain a score of one and be depicted by a deep green color. The results obtained from AGREE displayed in Table 3 indicate that the proposed spectrophotometric methods achieved an impressive score of 0.79, while the chromatographic methods mentioned in literature received a yellow representation with a comparatively lower scores of 0.49, 0.57 and 0.55 for the LC–MS/MS, UPLC and HPLC methods, respectively. This finding implies that compared to existing chromatographic methods, our proposed methods exhibit greener characteristics. The greenness evaluation of our proposed methods has also been compared with the reported spectrophotometric methods. The results showed that our proposed methods exhibited higher greenness scores compared to the reported spectrophotometric methods, indicating their superior eco-friendly characteristics. The major limitations in the greenness profile of the developed methods are related to items 1, 3, 7 and 10 of the AGREE analysis. Items 1 and 3 are related to the sampling site and position of the spectrophotometer; they require careful consideration as they should be performed off-site or in a controlled environment. This is why they were represented in yellow and orange colors, respectively. Furthermore, items 7 and 10 colored in yellow are associated with the amount of waste and types of reagents used. Although some of our reagents are bio-based such as water, they may still contribute to waste generation; therefore, proper management and disposal is necessary. In conclusion, the proposed spectrophotometric methods demonstrate superior eco-friendly characteristics compared to existing chromatographic methods and reported spectrophotometric methods based on greenness evaluation using the described metrics.

### Application to pharmaceutical formulation and greenness assessment

The developed methods utilized in this study effectively measured the concentrations of DTG and LMV in their respective pharmaceutical dosage forms. The obtained results were compared to those from a previously reported chromatographic method, revealing a high level of agreement (Table 4). To further assess any potential interference caused by excipients, the standard addition technique was employed, ultimately yielding excellent recoveries for both DTG and LMV (Table 4). Additionally, a comprehensive statistical analysis was conducted using *t*-tests and *F*-tests to compare the developed spectrophotometric methods with the reported chromatographic method. The results indicated that there is no significant difference between the two approaches, as evidenced by lower-than-expected t-values and F-values. A summary of these findings can be found in Table 4.

**Table 4:** Quantitative analysis of DTG/LMV in commercial Dovato^®^ tablets by the proposed methods and statistical comparison with the reported methods

DTG/LMV	%Recovery ± RSD							
	RD		^1^DD		CWT		Reported method ^6^	
	DTG	LMV	DTG	LMV	DTG	LMV	DTG	LMV
Dovato^®^ tablets^a^	99.36 ± 1.037	99.91 ± 0.729	99.11 ± 0.552	99.57 ± 0.700	99.21 ± 0.919	99.78 ± 0.659	99.67 ± 0.823	99.55 ± 0.849
Standard addition^b^	99.24 ± 0.609	100.99 ± 0.676	98.42 ± 0.311	100.94 ± 0.672	99.55 ± 0.536	100.28 ± 1.202		
*t*-test (2.101)^c^	0.756	1.029	1.809	0.067	1.192	0.692		
*F*-test (3.179)^c^	1.576	1.343	2.251	1.471	1.233	1.650		

^a^Average of ten determinations

^b^Average of three determinations

^c^The values in parenthesis are tabulated values of “t “and “F” at (P = 0.05)

## Conclusion and future directions

In conclusion, this research article presents the development and evaluation of three spectrophotometric methods for the simultaneous analysis of DTG and LMV in their binary mixtures. The methods, including RD, ^1^DD, and CWT, demonstrated significant potential in terms of simplicity, sensitivity, selectivity, and greenness profiles in quantifying the two antiretroviral drugs compared to reported spectrophotometric methods. In detail, the RD method offers a significant advantage as it can be applied at any two wavelengths throughout the entire ratio spectrum, without being affected by overlapped components. Moreover, the use of ^1^DD corrects for interferences and ultimately enhances the purity of analytical peaks. This is particularly advantageous when dealing with high levels of interfering compounds. On the other hand, the CWT improves signal-to-noise ratio and has multiple families available that cater to a wide range of applications, making it a highly promising approach for manipulating ratio spectra. Additionally, the developed methods offer a simpler and more cost-effective alternative to high-performance liquid chromatography. It is worth mentioning that the ^1^DD transcended the other methods in terms of accuracy, precision and sensitivity posing this method as a potential candidate for routine analysis of DTG and LMV in pharmaceutical formulations. The incorporation of the analytical eco-scale and AGREE for greenness evaluation further ensures that these spectrophotometric methods are more environmentally friendly than the chromatographic procedures. These results contribute to the field of pharmaceutical analysis by providing a comprehensive understanding of the greenness and practicality of spectrophotometric methods for the analysis of these two antiretroviral therapies.

Future studies could explore the application of these methods to analyze other antiretroviral drugs or different combinations of drugs. Additionally, it would be beneficial to investigate other related UV spectrophotometric techniques, such as diffuse reflectance spectroscopy, for the analysis of DTG and LMV in their solid forms. This technique has shown potential for direct analysis of solid samples [25], which could provide a more convenient and rapid method for quality control purposes.

## Data Availability

The data presented in this study are available on request from the corresponding author.
